# TECHNOLOGY-BASED INTERVENTIONS TO IMPROVE CAREGIVER WELL-BEING

**DOI:** 10.1093/geroni/igz038.2073

**Published:** 2019-11-08

**Authors:** Kaci Fairchild, Shirit Kamil-Rosenberg, Heather Taylor, Peter Louras, Blake Scanlon, Jonathon Myers, Jerome Yesavage

**Affiliations:** 1 Sierra Pacific MIRECC at VA Palo Alto, Palo Alto, California, United States; 2 VA Palo Alto, Palo Alto, California, United States; 3 Palo Alto University, Palo Alto, California, United States; 4 VA Palo Alto / Stanford University School of Medicine, Palo Alto, California, United States

## Abstract

Informal or unpaid care is the most common form of long-term care. Despite clear benefits for the care recipient, caregiving can have unintended physical and emotional consequences for caregivers. Traditional caregiver interventions are limited in scope, as they often focus on the emotional consequences of caregiving; however, the physiological effects of caregiving are equally deleterious to caregiver health. Exercise improves physical health, yet the demands of caregiving can limit participation in physical activity. Traditional gym-based interventions may not be feasible for many caregivers. Advances in technology present an opportunity to address these limitations, specifically in the areas of accessibility and acceptability. The Combined Online Assistance for Caregiver Health (COACH) program combines evidence-based skills training with physical exercise in a tablet-based intervention. Preliminary evidence for the physical and psychological benefits are promising; however, differential attrition rates are informative as to the acceptability of technology-based interventions among some caregivers.

